# Mitochondrial fusion‐ and fission‐related protein expression in the regulation of skeletal muscle mass

**DOI:** 10.14814/phy2.15281

**Published:** 2022-04-19

**Authors:** Daiki Nakano, Shuichi Machida

**Affiliations:** ^1^ 12847 Ritsumeikan University Kusatsu Japan; ^2^ Graduate School of Health and Sports Science Juntendo University Inzai Japan

**Keywords:** atrophy, Drp1, hypertrophy, mitochondrial quality control

## Abstract

Mitochondria in the skeletal muscle are essential for maintaining metabolic plasticity and function. Mitochondrial quality control encompasses the dynamics of the biogenesis and remodeling of mitochondria, characterized by the constant fission and fusion of mitochondria in response to metabolic stressors. However, the roles of mitochondrial fission or fusion in muscle hypertrophy and atrophy remain unclear. The aim of this study was to determine whether mitochondrial fusion and fission events are influenced by muscle hypertrophy or atrophy stimulation. Twenty‐six male F344 rats were randomly assigned to a control group or were subjected to up to 14 days of either plantaris overload (via tenotomy of the gastrocnemius and soleus muscles; hypertrophy group) or hindlimb cast immobilization (atrophy group). After 14 days of treatment, plantaris muscle samples were collected to determine the expression levels of mitochondrial fusion‐ and fission‐related proteins. Muscle weight and total muscle protein content increased following plantaris overload in the hypertrophy group, but decreased following immobilization for 14 days in the atrophy group. In the hypertrophied muscle, the level of activated dynamin‐related protein 1 (Drp1), phosphorylated at Ser616, significantly increased by 25.8% (*p* = 0.014). Moreover, the protein expression level of mitochondrial fission factor significantly decreased by 36.5% in the hypertrophy group compared with that of the control group (*p* = 0.017). In contrast, total Drp1 level significantly decreased in the atrophied plantaris muscle (*p* = 0.011). Our data suggest that mitochondrial fission events may be influenced by both muscle hypertrophy and atrophy stimulation, and that mitochondrial fission‐ related protein Drp1 plays an important role in the regulation of skeletal muscle in response to mechanical stimulation.

## Graphical abstract

In this study, we explored the role of mitochondrial dynamics‐related proteins, especially those involved in fission and fusion of the mitochondria, on muscle mass regulation in the context of hypertrophy and atrophy. To this end, we established a rat model of muscle hypertrophy using plantaris muscle overload with synergist ablation and a rat model of muscle atrophy by fixing the knee and ankle joints with casts for up to 14 days. Muscle weight and total protein content were increased and decreased by hypertrophy and atrophy, respectively, as expected. Moreover, the mitochondrial fission protein Drp1 was activated and translocated from the cytosol to the outer mitochondrial membrane under hypertrophy. Opposite effects were observed under atrophy.

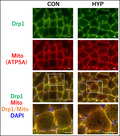

## 1 INTRODUCTION

The skeletal muscle is characterized by high plasticity, and regulation of muscle mass is primarily determined by protein metabolism. Chronic deprivation of mechanical stimulation to the muscles, such as that induced by denervation, bed rest, and immobilization, leads to muscle atrophy owing to a decline in protein synthesis and a relative increase in protein degradation (Krawiec et al., 2005; Lecker et al., 1999). In contrast, hypertrophic stimulation such as electric stimulation and overload activates protein synthesis. Although the link between mechanical signals and muscle mass regulation has been recognized, the underlying molecular mechanisms remain unknown.

Mitochondria in the skeletal muscle are essential for maintaining metabolic plasticity and function. Recent studies have shown that mitochondria play key roles in mediating muscle atrophy (Favoro et al., 2019.; Iqbal et al., 2013; Picard et al., 2015; Romanello & Sandri, 2015); Dysfunctional mitochondria release factors that induce apoptosis, such as cytochrome C and reactive oxygen species (ROS). These dysfunctional mitochondria are divided by mitochondrial fission, which plays a crucial role in the homeostatic control of muscle mass (Romanello & Sandri, 2015), and are then degraded by mitochondria‐selective autophagy (mitophagy) (Bonaldo & Sandri, 2013; Romanello & Sandri, 2015).

Mitochondrial quality control encompasses the dynamic processes of biogenesis and remodeling, comprising mitochondrial fusion and fission events. Mitochondrial fusion is regulated by mitofusin 1/2 (Mfn1/2) in the mitochondrial outer membrane and optic atrophy 1 (Opa1) in the mitochondrial inner membrane, whereas fission events are regulated by dynamin‐related protein 1 (Drp1), mitochondrial fission factor (Mff), and fission protein 1 (Fis1) (Romanello et al., 2010). In a mechanistic study, Romanello et al. (2010) showed that muscle‐specific knockdown of Drp1 resulted in muscle wasting with functionally abnormal mitochondria. In contrast, acute hypertrophic electric stimulation was shown to activate Drp1 (Kitaoka et al., 2015). These studies and others (e.g., Helle et al., 2017) demonstrate that mitochondrial dynamics–related proteins may be essential in muscle mass regulation. However, the specific roles of mitochondrial fusion and fission events in response to chronic mechanical stimuli remain unclear. Accordingly, the aim of this study was to determine the influence of muscle hypertrophic or atrophic stimulation on mitochondrial fusion and fission events in a rat model.

## 2 MATERIALS AND METHODS

### 2.1 Animals and surgeries

Male F344 rats (13 weeks old) obtained from Japan SLC (Shizuoka, Japan) were used in this study. The rats were housed with free access to food and water until the morning of the experiment. All animals were maintained in a temperature‐controlled environment of 22–24°C with a 12‐h light‐dark cycle. The rats were randomly assigned to the synergist ablation (hypertrophy group; *n* = 8), casting (atrophy group; *n* = 9), or control (*n* = 9) groups. In the hypertrophy group, the gastrocnemius and soleus muscles from both legs were removed under 1.5% isoflurane anesthesia (1.5 L/min) and overloading of the plantaris muscle was induced by excising the distal half of both the gastrocnemius and soleus (Goodman et al., 2011) for 14 days. Animals in the control and atrophy groups underwent incision and suturing, but without removal of tissues as a sham operation. The rats in the control group were maintained with no further treatment for 14 days. In the atrophy group, the knee (in a 75° bent position) and ankle (in a 30° bent position) joints were fixed by casting for 14 days as previously described (Booth & Kelso, 1973) under 1.5% isoflurane anesthesia (1.5 L/min).

The Institutional Review Board and Ethics Committee of Juntendo University approved this study (approval no. H30‐04). All experiments were performed according to the American Physiological Society's Guiding Principles for the Care and Use of Animals.

### 2.2 Tissue extraction

After each treatment, the animals were euthanized under 1.5% isoflurane anesthesia (1.5 L/min) and the complete plantaris muscle was removed. Half of the muscle was rapidly frozen in liquid nitrogen and stored at –80°C until biochemical analysis, and the other half was frozen in isopentane and cooled with liquid nitrogen until immunohistochemical staining.

### 2.3 Western blotting

Plantaris muscle samples were homogenized in homogenization buffer (RIPA Buffer, Thermo Fisher Scientific, Waltham, MA, USA), PhosSTOP EASY pack (Roche, Basel, Switzerland), and Halt Protease Inhibitor Cocktail (Thermo Fisher Scientific). The homogenates were centrifuged at 14,000 × *g* for 10 min at 4°C, the supernatants were removed, and the protein concentration in each sample was quantified using a bicinchoninic acid assay kit (Thermo Fisher Scientific). Equal amounts of protein were resolved by sodium dodecyl sulfate‐polyacrylamide gel electrophoresis using 10% gels and transferred to polyvinylidene difluoride membranes, and the transfer efficiency was confirmed with Ponceau S solution. Western blotting was performed using primary antibodies against the following proteins: Drp1 (1:1000; cat. no. ab56788; Abcam, Cambridge, UK) and Drp1 phosphorylated at Ser616 (phosphor‐DRP1^Ser616^; 1:1000; cat. no. 3455; Cell Signaling Technology, Danvers, MA, USA), Mff (1:5000; cat. no. ab129075; Abcam), Fis1 (1:2000; cat. no. ab96764; Abcam), Mfn2 (1:5000; cat. no. ab124773; Abcam), Opa1 (1:1000; cat. no. 612606; BD Transduction Laboratories, Franklin Lakes, NJ, USA), and Parkin (1:1000; cat. no. ab77924; Abcam). The membranes were then incubated with horseradish peroxidase‐linked secondary antibody (1:5000 for anti‐rabbit IgG; cat. no. ab6741; Abcam or for anti‐mouse IgG; cat. No. NA931; GE Healthcare UK Limited, Buckinghamshire, UK) and visualized using a chemiluminescent substrate (cat. no. WSE‐7120L; ATTO, Tokyo, Japan). Blots were scanned, and band intensities were quantified using ChemiDoc Touch Imaging System (Bio‐Rad, Hercules, CA, USA) and Image Lab (version 6.0.0; Bio‐Rad).

### 2.4 Immunohistofluorescence staining

To determine the localization of Drp1, frozen sections (10 μm) from the plantaris muscles were labeled with antibodies targeting the mitochondrial marker ATP5A (1:100; cat. no. ab176569; Abcam) and Drp1 (1:500; cat. no. ab56788; Abcam). Anti‐rabbit IgG Alexa546 (1:500; cat. no. A11010; Thermo Fisher Scientific) and anti‐mouse IgG (1:200; cat. no. FI‐2001; Vector Laboratories, Burlingame, CA, USA) were used as secondary antibodies. Coverslips were mounted onto glass slides using mounting medium with DAPI (cat. no. H‐1500; Vector Laboratories). For imaging, the sections were visualized using a BZ‐X800 microscope (Keyence, Osaka, Japan) using a 20 × objective lens. Optical sectioning technology, which allows the sectioning of images for determining localization, was used in a previous study (Nishiyama et al., 2019) and was also used in the present study with the BZ‐X800 microscope.

### 2.5 Statistical analysis

Data are presented as the mean ±standard deviation. *T*‐test was used to compare the hypertrophy and atrophy groups with the control group. Differences were considered significant at *p* values less than 0.05. Analyses were performed using SPSS version 24.0 (IBM Corp., Armonk, NY, USA).

## 3 RESULTS

### 3.1 Effects of hypertrophic stimulation on muscle size and total protein content

**FIGURE 1:**
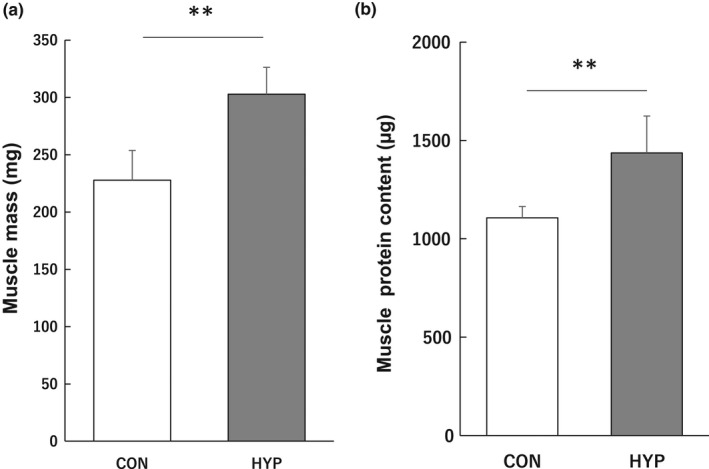
Effects of hypertrophic stimulation of the plantaris muscle on muscle mass (a) and total protein content (b). Data represent the means ±standard deviations (*n* = 8–9 rats/group). Differences in muscle mass and total protein content were assessed by *t*‐test. Significant differences with the control group are indicated by **p < 0.01. Abbreviations: CON, control group; HYP, hypertrophy group

Hypertrophy via synergistic ablation induced a significant increase in plantaris muscle weight (33.0%; Figure 1a) and total protein content (29.8%; Figure 1b) compared with those of the control group.

### 3.2 Effects of hypertrophic stimulation on mitochondrial dynamics

**FIGURE 2:**
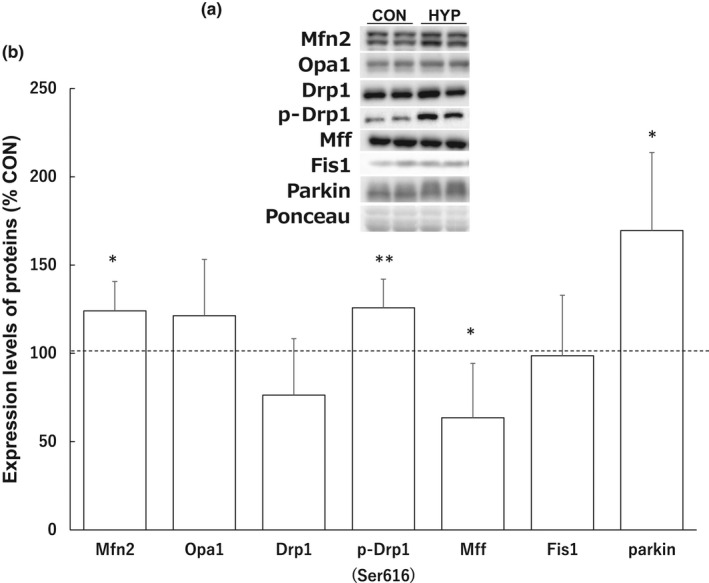
Effects of hypertrophic stimulation of the plantaris muscle on mitochondrial fusion and fission‐related proteins. (a) Representative western blots of proteins. (b) Expression levels of mitochondrial fusion and fission‐related proteins. Protein expression is expressed as a relative value with the value of the control group taken as 100. Data represent the means ± standard deviations (*n* = 8–9 rats/group). Differences in expression levels of proteins were assessed by *t*‐test. Significant differences with the control group are indicated by **p < 0.01. Abbreviations: CON, control group; HYP, hypertrophy group

**FIGURE 3:**
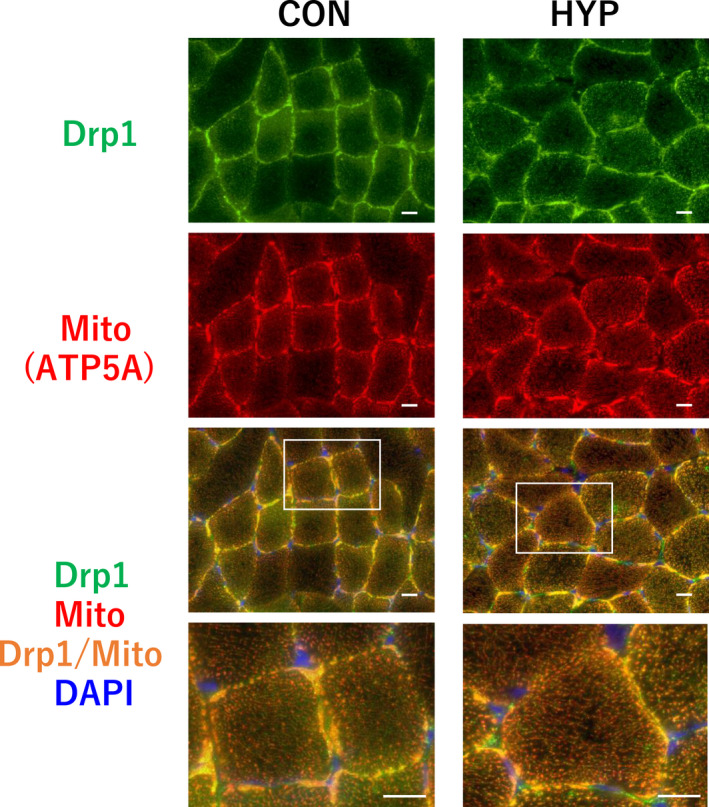
Effects of hypertrophic stimulation of the plantaris muscle on co‐localization of Drp1 and mitochondria (ATP5A). Immunofluorescence images for the control group (Left). Immunofluorescence images for the hypertrophy group (Right). Scale bar = 20 μm. Upper row: Drp1; second row: mitochondria (ATP5A); third row: merged image; bottom: enlarged view of merged image. Abbreviations: CON, control group; HYP, hypertrophy group

As shown in Figure 2, hypertrophic stimulation did not alter the expression of Drp1 and Fis1, although the protein level of Mff significantly decreased by 36.5% (*p* = 0.013) in the hypertrophied muscle. Moreover, the level of Mfn2 significantly increased by 24.0% (*p* = 0.048) in the hypertrophy group. Parkin, ubiquitinate, and Mff turnover significantly increased by 69.5% (*p* = 0.04) in the hypertrophied muscle. In contrast to total Drp1 protein, the level of phospho‐Drp1^Ser616^ was significantly increased by 25.8% (*p* = 0.009) in the hypertrophy group, indicating increased Drp1 activation. Immunohistochemical staining of cross‐sections of the plantaris muscles showed that endogenous Drp1 was localized in the cytosol of the control muscle, but on the mitochondria of hypertrophied muscle fibers (Figure 3).

### 3.3 Effects of atrophic stimulation on muscle size and total protein content

**FIGURE 4:**
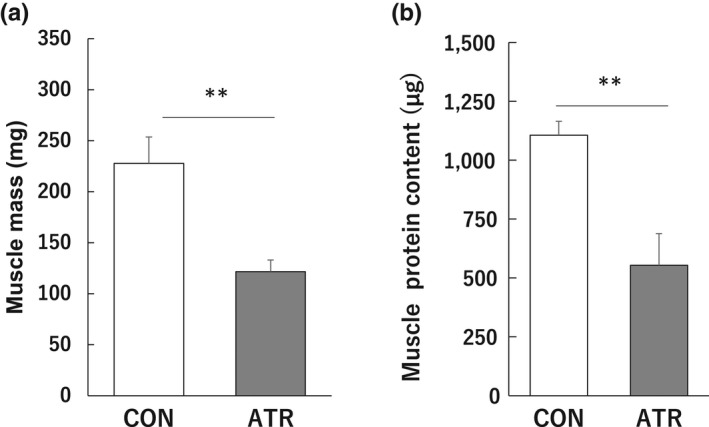
Effects of atrophic stimulation of the plantaris muscle on muscle mass (a) and total protein content. (b) Data represent the means ± standard deviations (*n* = 8–9 rats/group). Differences in muscle mass and total protein content were assessed by *t*‐test. Significant differences with the control group are indicated by **p < 0.01. Abbreviations: CON, control group; ATR, atrophy group

Plantaris muscle weight (Figure 4a) and total protein content (Figure 4b) decreased by 46.6% and 49.9% in the atrophy group compared with that in the control group.

### 3.4 Effects of atrophic stimulation on expression of mitochondrial dynamics

**FIGURE 5:**
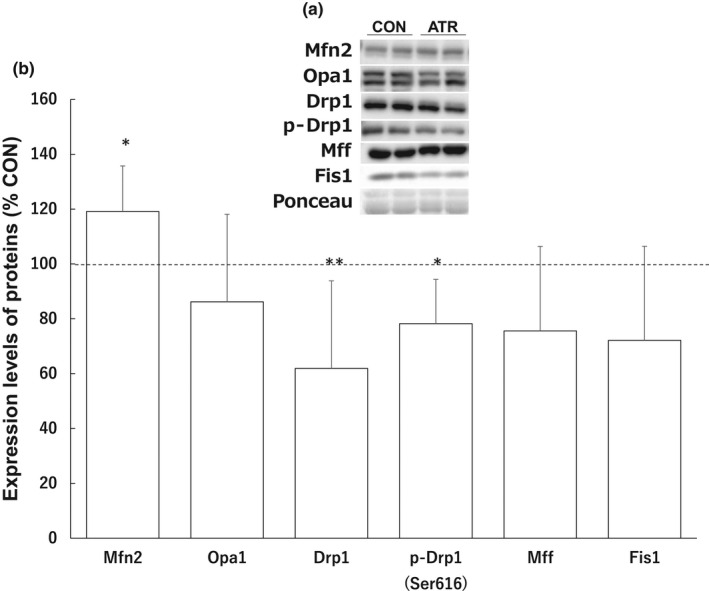
Effects of atrophic stimulation of the plantaris muscle on mitochondrial fusion and fission‐related proteins. (a) Representative western blots of proteins. (b) Expression levels of mitochondrial fusion and fission‐related proteins. Protein expression is expressed as a relative value with the value of control group taken as 100. Data represent the means ± standard deviations (*n* = 8–9 rats/group). Differences in expression levels of proteins were assessed by *t*‐test. Significant differences with the control group are indicated by **p < 0.01. Abbreviations: CON, control group; ATR, atrophy group

**FIGURE 6:**
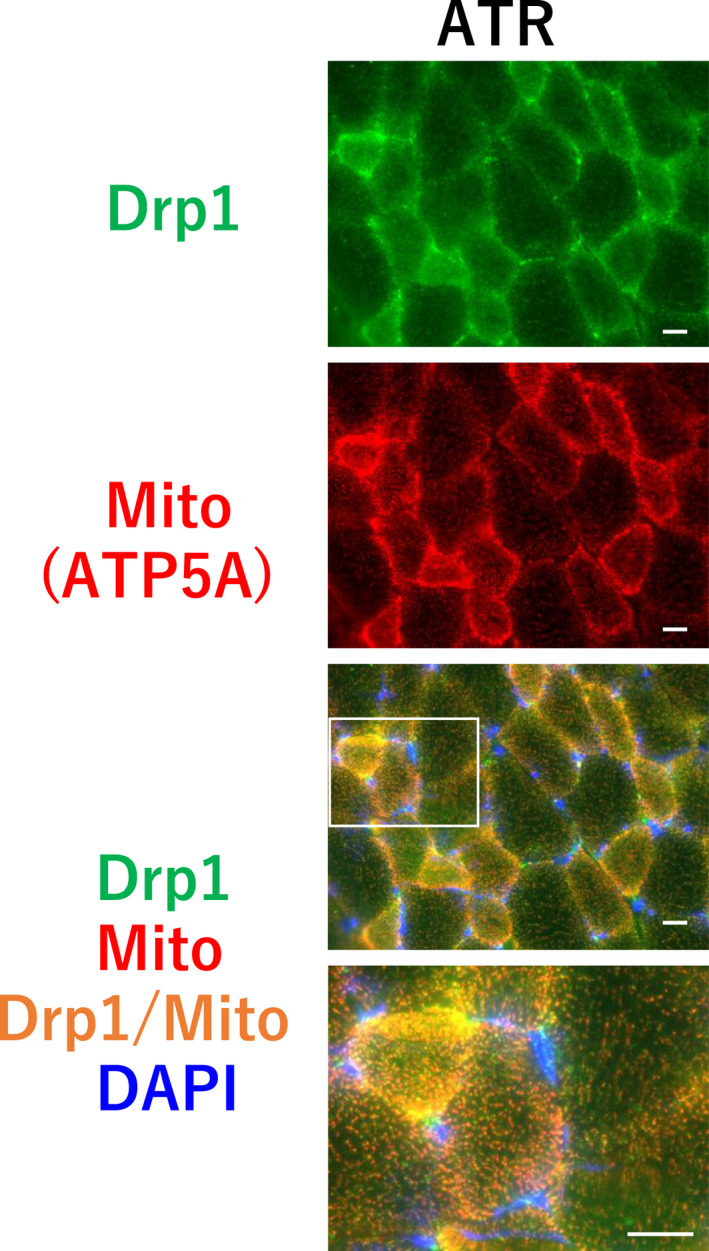
Effects of atrophic stimulation of the plantaris muscle on co‐localization of Drp1 and mitochondria (ATP5A). Scale bar = 20 μm. Upper row: Drp1; second row: mitochondria (ATP5A); third row: merged image; bottom: enlarged view of merged image. Abbreviations: ATR, atrophy group

As shown in Figure 5, atrophic stimulation did not alter the expression of Opa1, Mff, and Fis1. However, the protein level of Drp1 and phospho‐Drp1^Ser616^ significantly decreased by 38.1% and 21.8%, respectively, in the atrophied plantaris muscle, while Mfn2 significantly increased by 19.1% (*p* = 0.028). Immunohistochemical staining of cross‐sections of the plantaris muscle showed that Drp1 was localized in the cytosol of muscle fibers in both the control (Figure 3) and atrophy groups (Figure 6).

## 4 DISCUSSION

In this study, we determined the expression of mitochondrial fusion‐ and fission‐related proteins in skeletal muscle following 14 days of overload or casting. We used a synergist ablation model to induce muscle hypertrophy, which has previously been found to increase muscle mass and the muscle fiber cross‐sectional area (Bodine et al., 2001; Kano et al., 1997; Terena et al., 2017). In this study, 14 days of tenotomy was sufficient to induce hypertrophy, accompanied by an increase in muscle mass and total protein content in skeletal muscle following 14 days of overload. Although chronic mechanical overload (i.e., hypertrophy) did not alter the expression of total Drp1, activated Drp1 (phospho‐Drp1^Ser616^) levels were significantly increased and the level of Mff was significantly decreased in the hypertrophied muscle. In contrast, total Drp1 levels were significantly decreased in the atrophied muscles. Our data suggest that mitochondrial fission events may be influenced by both chronic muscle hypertrophy and atrophy stimulation.

Drp1 is present in the cytosolic fraction, and then moves to the mitochondrial outer membrane when a mitochondrial fission event is required (Romanello et al., 2010). This step requires activation of Drp1 via its phosphorylation at the Ser616 residue (Taguchi et al., 2007). Therefore, to clarify the role of Drp1 in the regulation of muscle size, including atrophy and hypertrophy, verification of the expression level of phospho‐Drp1^Ser616^ and localization of Drp1 is required. We qualitatively confirmed increased localization of Drp1 at the mitochondria under the hypertrophy condition. Tarpey et al. (2017) reported that endurance training promotes mitochondrial quality control by increasing phospho‐Drp1^Ser616^ levels. Moreover, Kitaoka et al. (2015) found that the expression level of Drp1 did not change in hypertrophied muscles subjected to electric stimulation, whereas activation of Drp1 was promoted following hypertrophic stimulation, in line with our results. These studies suggest that activation (localization) of Drp1 plays an important role in skeletal muscle adaptation to mechanical stimulation. Interestingly, the level of Mff, localized in the mitochondrial outer membrane, decreased in skeletal muscle following 14 days of overload. Lee et al. (2019) demonstrated that Mff was ubiquitinated by Parkin; we also confirmed that the expression level of Parkin decreased in the hypertrophied plantaris muscle. This suggests that hypertrophic stimulation promotes the ubiquitination of Mff for turnover by Parkin.

We used a casting model as the traditional method to induce muscle atrophy (Booth & Kelso, 1973). Casting decreases muscle mass and the cross‐sectional area of muscle fibers, and promotes protein degradation (Nozaki et al., 2020), which were confirmed in the current study. During muscle atrophy, excessive ROS production damages mitochondrial DNA, resulting in mitochondrial dysfunction (Muller et al., 2007; Wei, 1998). Mitochondrial fission and mitophagy are important for the removal of dysfunctional mitochondria (Bonaldo & Sandri, 2013; Romanello & Sandri, 2015). Favaro et al. (2019) reported that muscle‐specific loss of Drp1 promotes muscle wasting. However, another study with a lower level of Drp1 depletion (i.e., 40%) failed to observe atrophy (Moore et al., 2019). Drp1 has been increasingly considered, but conflicting results have been reported. Data are especially lacking on the role of Drp1 in skeletal muscle mass after the developmental phase. In the current study, western blot analysis indicated that the total Drp1 level significantly decreased in the atrophied muscle after 14 days of hindlimb cast immobilization, which induced severe muscle atrophy (46.6% decrease in muscle mass). Dulac et al. (2020) recently reported that knockdown of Drp1 for 4 months in adult mouse skeletal muscle resulted in severe muscle atrophy (40–50%). Thus, these data suggest that Drp1 is important for the maintenance of adult skeletal muscle mass.

In conclusion, our data suggest that mitochondrial fission events are influenced by adult muscle hypertrophy or atrophy stimulation. However, our study only reported the expression patterns of mitochondrial fission‐related proteins, and the role of the mitochondrial fission factor Drp1 in response to chronic mechanical stimuli remains unclear. Therefore, further studies are required to clarify the molecular mechanisms that modulate the effects of Drp1‐mediated mitochondrial quality control on muscle overload‐induced hypertrophy or immobilization‐induced atrophy. Moreover, these findings have implications for improving quality of life and recovery in individuals with chronic muscle atrophy or hypertrophy, and offer new directions for mechanistic research in the field.

## CONFLICT OF INTEREST

The authors have no conflict of interest to declare.
